# Poorer self-reported mental health and general health among first year upper secondary school students do not predict school dropout: a five-year prospective study

**DOI:** 10.3389/fpsyg.2024.1304314

**Published:** 2024-02-08

**Authors:** Charlotte Bjørnskov Goll, Tore Sørlie, Oddgeir Friborg, Karl Ottar Ottosen, Rannveig Grøm Sæle

**Affiliations:** ^1^Department of Clinical Medicine, UiT The Arctic University of Norway, Tromsø, Norway; ^2^Department of Mental Health and Substance Abuse, University Hospital of North Norway (UNN), Tromsø, Norway; ^3^Department of Psychology, UiT The Arctic University of Norway, Tromsø, Norway

**Keywords:** school dropout, mental health, general health, academic performance, upper secondary school

## Abstract

**Introduction:**

Education is important for socioeconomic, work and health status; thus, dropping out of secondary school is of major concern. In Norway, 1 out of 5 is dropping out from upper secondary education. Academic performance is a known predictor for dropout, but the role of mental and general health status is studied less.

**Methods:**

By use of student data collected during the first school year we examined the accumulated risk of school dropout over 5 years. Students entering upper secondary school in a North-Norwegian region (Troms County) completed a comprehensive questionnaire during August 2010 (*N* = 1,676, 69% response rate). The contribution of mental and general health problems in predicting five-year dropout was of primary interest, adjusted for demographics and academic performance.

**Results:**

One-third of the students had dropped out after 5 years. A logistic regression analysis showed no significant effect of mental and general health problems on dropout. Among the covariates, higher grades from lower secondary education reduced the chance of dropping out (OR = 0.31; *p* < 0.001). Subgroup analyses showed that students in the vocational track reported poorer mental and general health, compared to students in the general track, but this difference was not related to dropout. General track students were also less likely to drop out than vocational track students (OR for dropout 0.66; *p* < 0.05).

**Discussion:**

In conclusion, lower grades from lower secondary education represented a warning flag for school dropout during upper secondary education whereas mental health issues were not.

## Introduction

Dropout from upper secondary education is a major concern in Norway, and in the rest of Europe, as it increases the risk of unemployment, poorer mental health and even mortality (De Ridder et al., 2013; Markussen, 2017; Hale and Viner, 2018; OECD, 2022; Hetlevik et al., 2023). In technologically advanced information societies, higher education is a key requisite for success in the labour market (Halvorsrud, 2017; Markussen, 2017; OECD, 2022). The expectations of acquiring education are high, both among the students and in the society, as employers of today are less willing to accept unskilled labour. The high number who continue secondary schooling after finishing lower secondary – 98% in Northern Norway (Norwegian Directorate for Education and Training, 2023) is perhaps a reflection of this trend. However, in Norway, about 1 in 5 students do not complete upper secondary schooling within 5 years. In Northern Norway, where this study was conducted, this problem is even more pronounced, with 1 in 4 (25%) not completing within 5 years (Statistics Norway, 2023).

**Figure 1:**
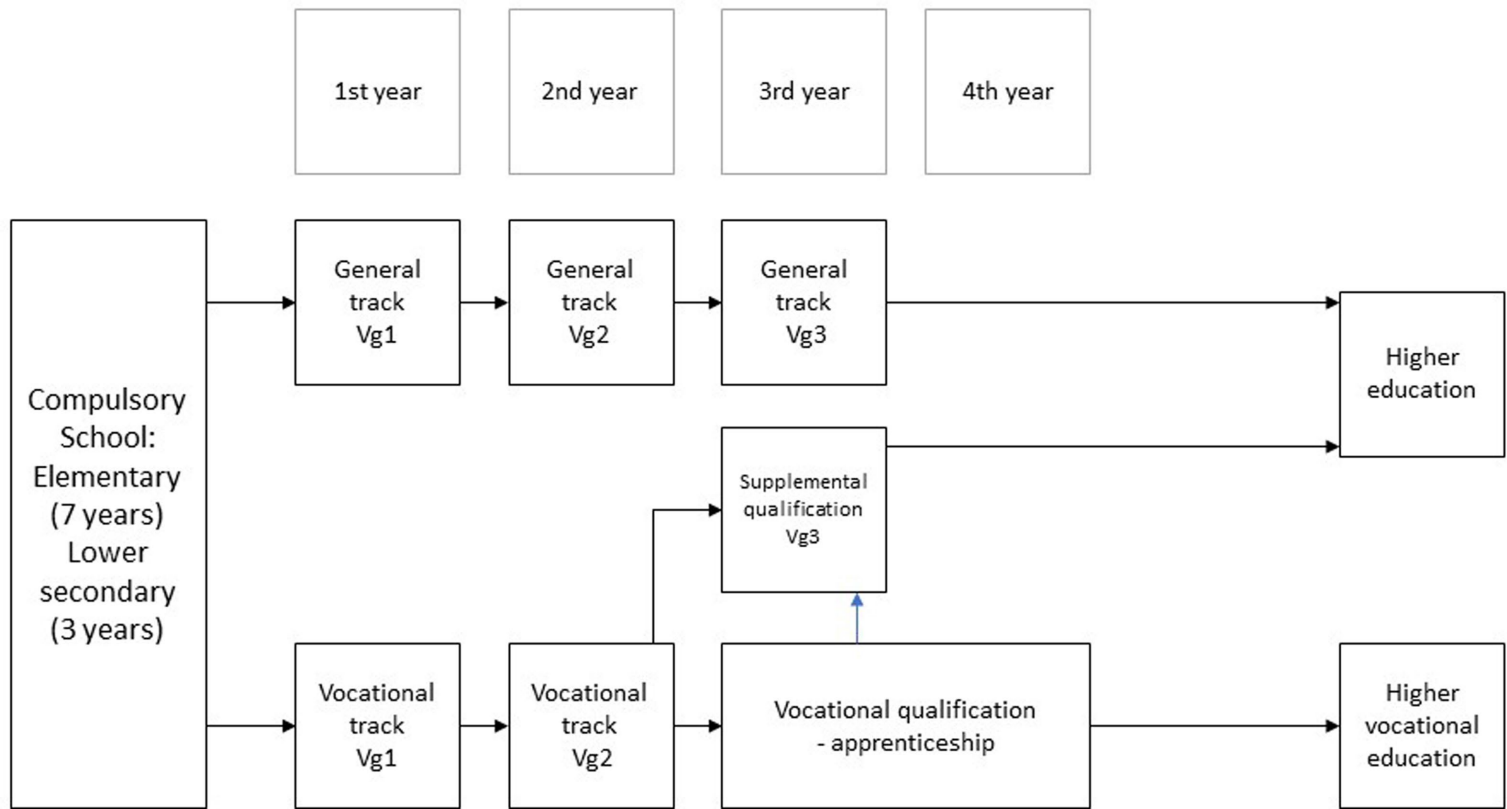
Norwegian upper secondary school (based on Vilbli, 2024).

Upper secondary school in Norway includes two main tracks: The general track preparing students for higher education, and a vocational track providing students with a vocational qualification enabling them to enter the labour market directly after finishing school (Vilbli, 2024). Both tracks have a theoretical foundation, but theory is less pronounced and more combined with practical training in the vocational track (Norwegian Directorate for Education and Training, 2023). A graphical representation of the Norwegian school system is provided in Figure 1.

A variety of individual, family, socio-economic and school related factors go along with dropout (Rumberger and Lim, 2008; Markussen et al., 2011; Dæhlen, 2017). In the following sections, we summarize the empirical background for the inclusion of the current study variables.

Poor *general health* may limit the opportunities for participation in the labour market as well as in the society in general (Curnock et al., 2016; Thern et al., 2017; OECD, 2022). It is well known that there is a social gradient in health (WHO, 2013a) and poor health, school dropout and receiving social benefits are closely related to each other (De Ridder et al., 2012, 2013). About 10% of the Norwegian population receive disability benefits, and the percentage is increasing in the younger ages (18–44) (NAV, 2023b). At the age of 30, approximately 7% of the population receive such benefits.

In the younger groups receiving disability benefits, more than 60% had *mental health* diagnoses (NAV, 2023a). The most common mental health issues among adolescents in general (WHO, 2021). In Norway, mental health disorders affect about 15–20%, and according to Norwegian Institute of Public Health approximately half of these needs (NIPH, 2018). According to WHO, depression is one of the largest causes to disability also in (WHO, 2013b, 2021) and Patel et al. (2007) connects the inflicted mental health burden in young people to lower educational achievements and substance abuse. Previous studies have shown that poor mental health is associated with dropout in upper secondary school (Gustafsson et al., 2010; Hjorth et al., 2016), and particularly among students in the vocational track (Hjorth et al., 2016). In addition, poor mental health relates to other health conditions, such as *disturbed sleeping* (Zhang et al., 2017) and *eating disturbances* (Allen et al., 2013), and these variables were also investigated.

Adolescence is a highly transformational period involving bodily changes, identity formation and general worry. It is characterized by uncertainty, many choices, new experiences and finding a role in the society, all while navigating between often contrasting opinions and expectations from family and friends. Having a stronger *sense of coherence* (SOC; i.e. meaningfulness, comprehensibility and manageability) may be an advantage for adolescents in order to formulate and maintain plans that are in accordance with their own needs and preconditions, despite external stressors or strains that otherwise would increase the risk of dropout. Since a higher SOC is also protective against both poor mental health and poor health in general (Kouvonen et al., 2010; Super et al., 2014; Mittelmark et al., 2017), it is a relevant concept to examine in the context of school dropout.

Students achieving lower grades in their courses have a considerably higher risk for dropout compared to students achieving higher grades (Bowers, 2010). *Previous grades* also predict dropout (Casillas et al., 2012; Statistics Norway, 2023). According to Statistics Norway (2023), the completion rate among upper secondary students is approximately 98% among students with top grades from lower secondary school, while it is only approximately 42% among those in the lowest grade range.

Students experiencing *learning problems* tend to have lower educational attainment and aspirations than their non-afflicted peers (Wagner et al., 2005; Kortering et al., 2010; Irvin et al., 2011), which contribute to the dropout problem (Korhonen et al., 2014; Gubbels et al., 2019). As adults, they earn less – an effect that is mediated by attainment levels (McLaughlin et al., 2014), which underscores the importance of school completion. Gender may play a modifying role, as girls with learning problems attain more education than boys with similar problems (McLaughlin et al., 2014).

The dropout-rate in the vocational *track* is higher than in the general track (Statistics Norway, 2023) and Hjorth et al. (2016) showed that mental health measured by the Short-Form Health Survey (SF-12) prior to their dropout, was associated with dropout in the vocational track. A higher percentage of males are in the vocational track (Statistics Norway, 2023). This combined with higher risk of learning problems (see above) and lower academic achievement (Markussen et al., 2011; Sæle et al., 2016) may be one explanation of the higher dropout-rate in males. Derdikman-Eiron et al. (2011) showed that even though females had higher depression and anxiety scores, this did not influence academic performance as it did for males. The *gender* differences in school performance and dropout need to be explored further.

Also, other factors may be relevant for our context. Psychosocial factors, such as being *bullied* and receiving *social support*, influence both mental health (Reid et al., 2016) and school-related problems (Bania et al., 2016). Motivation is a central variable underpinning learning processes (Dweck, 1986). One aspect of motivation is the type of goals (Lockwood et al., 2002). Examples of such goal types are *prevention goals*, which motivates avoidance of failure, and *promotion goals*, which motivates towards achieving success.

Objectives: This study investigated empirically selected predictors – measured at the beginning of the first study year – of dropout from upper secondary school 5 years later as recorded by the county’s registry. The present study use data from the longitudinal research project “young will,” aiming to investigate predictors and consequences of dropout through data collections during a ten-year period.

Based on the above summary, we expected the following: dropout rates would be highest among (1) male students, (2) students in vocational track, (3) students with more learning problems, (4) students with poorer grades from lower secondary education, and (5) students with poorer general health, more internalized mental health problems, more eating problems, less life satisfaction and less sense of coherence.

We also examined if there were associations between dropout and feminine or masculine personally traits, total sleep time, motivation patterns, social support, and SES.

Finally, we examined if scores on self-reported mental and general health changed significantly during the upper secondary school years.

## Methods

### Participants and procedures

All students entering the first year of upper secondary school in Troms County in the autumn of 2010 were invited to participate. Recruitment was done through school personnel, who informed the students about the study. Of the 2,434 students, 1,676 (69% response rate) consented in written and completed a questionnaire in class during August, just after study start. For students under the age of 16, parental consent was collected. A follow up study was conducted 30 months later through e-mail. The attrition of participants was very high (*N* = 660, 39% participated) despite two reminders, which precluded use of these data in the dropout analyses.

The sample consisted of 48% females and the mean age was 16.7 (SD = 1.3). The majority were ethnic Norwegians (94%), whereas 4.7% identified as Sami, Kven or Finnish, and 5.5% as other.

The study was approved by the Regional Committee of Medical and Health Research Ethics in North Norway (ref no: 2010/1503).

### Measures and variables

Student status after 5 years was coded as: (0) completed and passed upper secondary, (1) completed, but not passed, (2) still in school, (3) not registered on any of the county’s schools, and (4) dropout. For the outcome analysis, only the first and the last category was retained and recoded as 0 – completed/passed (coded 0 initially) and 1 – dropout (coded 4 initially). This left us with 1,447 cases, of which 473 (32.7%) had dropped out.

The questionnaire consisted of a range of measures considered relevant for adolescents in school. In this paper, we have focused on demographics, health, personality and educational variables. Most of the instruments used in our study have earlier been used and validated in studies of adolescents and young adults in Norwegian and international settings (Bøe et al., 2012; De Ridder et al., 2013; Bania et al., 2016; Markussen, 2017) (see below for detailed descriptions of instruments used in this paper). Data on student dropout, lower secondary grade point average (GPA), sex and birth dates, were collected through the county’s registry.

HSCL-5, a short version of HSCL-10 (Hopkins Symptom Checklist), has been used earlier in studies of adolescents (Strand et al., 2003; Myklestad et al., 2012). HSCL-5 is a self-rating scale with five items that accesses internalizing problems during the past 2 weeks. Strand et al. (2003) found that a mean score above 2 can indicate a mental disorder, and we collapsed the variable into two categories (below and above this cut-off). The internal consistency (Cronbach‘s α = 0.85) in our study corresponds to previous studies (Tambs and Moum, 1993; Strand et al., 2003).

The Satisfaction with Life Scale (SWLS) is developed by Diener et al. (1985), includes five items and is used widely in population surveys, also among adolescents in Norway (Moksnes et al., 2014). Cronbach’s α = 0.89 and corresponds to earlier studies (Pavot et al., 1993; Moksnes et al., 2014).

Total sleep time in workdays was measured by the Munich ChronoType Questionnaire (MCTQ) (Roenneberg et al., 2003) translated to Norwegian. The actual sleep time in hours was calculated as: Bedtime (time going to bed) to wake-up time subtracted the time it took to fall asleep. Roenneberg et al. (2004) used the MCTQ in a large population survey including adolescents.

General health was measured by Self-rated health (SRH), which is a single-item variable used in several adolescent surveys (Idler and Benyamini, 1997; Breidablik et al., 2009). The response categories were 1-very good, 2-good, 3-not so good, and 4-poor, and we dichotomized these into two categories (very good/good and not so good/poor) as in a study of De Ridder et al. (2012).

The Eating Disturbance Scale (EDS-5) (2001) estimates general eating problems. EDS-5 consists of five-items and uses a seven-point Likert scale, earlier used in a survey among adolescents in Norway (Heradstveit et al., 2019). Cronbach’s α in this study = 0.79, which corresponds to findings in studies of Rosenvinge et al. (2001) and Heradstveit et al. (2019).

The Sense of Coherence scale is based on Antonovsky’s salutogenic model of coping with stressors (Antonovsky, 1993). It consists of 13 items, mapping meaningfulness, comprehensibility and manageability. SOC has previous been used by Moksnes et al. (2013) in an adolescent population. Cronbach’s α = 0.84, corresponding to the original reports and similar studies (Moksnes et al., 2014). We reversed the scores before including the scale in the analyses, making it correspond with the direction of the other included scales (higher scores = poorer sense of coherence).

Norwegian students normally start in upper secondary school directly after compulsory school, which means they are 15–16 years old. The age range in this sample was, however, 15–31, which means that some of the participating students were considerably older. We made a dichotomous variable with students aged 15 and 16 years old at the time of data collection in one category (students born in 1994, plus three younger students, born in 1995; 84% of the sample) and older students in the other.

We included measures of socio-economic status (SES), and asked them “*Compared to others in Norway, I consider our family’s economy as*; 1 – poor, 2 – average, 3 – good or 4 – very good.” The question was previously used in the Bergen Child Study (Bøe et al., 2012) and in Young-HUNT (HUNT, 2013), and when asked to parents, it correlated moderately with actual monetary income (*r* = 0.59) (Bøe et al., 2012). Goodman et al. (2001) have shown that adolescents’ perception of the family economic status correlate highly with objective social status (*r* = 0.72–0.79). We also asked questions regarding parental educational level, but these contained a large amount of “I do not know”-responses and had to be discarded from analyses, leaving us with the one item measuring SES.

GPA was calculated as the average of all grades recorded the last year of lower secondary school. Possible scores range from 0 (poorest) to 6 (best), with 2 as the pass mark. Mean GPA for the sample was 4.1 (SD = 0.79; range 1.56–5.94). In 19.2% of the records, GPA was missing, for various reasons: Some students may have been enrolled at upper secondary with other qualifications than GPA (like age), some students had a Steiner school background without grades, and some students had grade exemptions due to learning or behavioural disabilities.

We assessed social support by six questions, for example “*Do you have friends appreciating you,”* and *“Do you ever feel lonely.”* Items were rated 1 to 5. Some of the items have previously been used in several large Norwegian population studies including adolescents (Breidablik and Meland, 2001; HUNT, 2013; Tromsøundersøkelsen, 2013). A principal component analysis indicated a single component with an eigenvalue >1 (λ = 2.46, *R*^2^ = 41%). The total score range was 6–30 (Cronbach’s α = 0.69).

One item was used to assess bullying: “*I am bullied or harassed at my school”* (range 1–6). The item has previously been used in the young-HUNT study (HUNT, 2013).

The Personal Attributes Questionnaire (PAQ; Spence and Helmreich, 1978) measures the presence of personality traits with 24 items belonging to three subscales: Masculinity (M), femininity (F) and masculinity-femininity (M-F) (Helmreich et al., 1981). The masculine and feminine subscales represent traits that are seen as desirable for both sexes, but most prevalent in one or the other (e.g., *competitive* (M) and *understanding* (F)). The last subscale represents traits that are considered desirable only for one sex (e.g., submissive (F) and aggressive (M)). In our sample, this subscale had a Cronbach’s alpha as low as 0.46 and was not included in the analyses (see also Sæle et al., 2016). The masculinity and femininity subscales had a presentable Cronbach’s α = 0.73 and 0.82, respectively compared with the originally scale (0.85 and 0.82 respectively) (Spence and Helmreich, 1978; Sæle et al., 2016).

To assess motivational goals, we used the Promotion/Prevention scale (Lockwood et al., 2002), which builds on Regulatory focus theory (Higgins and Fowler, 1997). The scale has been used earlier in a sample of university students (Lockwood et al., 2002). A promotion focus involves being motivated by achieving a desirable outcome, while a prevention focus involves being motivated by avoiding a non-desirable outcome. Sample items are “*I typically focus on the success I hope to achieve in the future”* (promotion) and “*I frequently think about how I can prevent failures in my life”* (prevention). In our sample, Cronbach’s α was 0.88 for promotion and 0.79 for prevention, which corresponds to the original study (Lockwood et al., 2002).

Reading and writing problems, or literacy problems, were measured by a short scale addressing current and previous difficulties with decoding, reading comprehension, spelling, and written expression. The psychometric properties of the scale, as reported in a previous paper (Sæle et al., 2016), are satisfactory. Sample items are “*Do you have reading difficulties?”* and “*Do you make many writing mistakes?”* Cronbach’s α = 0.84.

Among the last variables included in the analyses were track. The students are normally enrolled either in general or vocational track. However, 11 students were enrolled in an alternative track; a track with schedules and teaching individually adapted. Since these students represented a very small group, they were treated as missing on the Track variable, leaving only the two main categories. In addition, we included ambitions (measured by having plans for further education or not).

### Data analyses

Two cases were excluded because they were multivariate outliers (logit residuals were − 20.96 and − 17.13), leaving 1,674 cases eligible for analysis. We excluded further 227 students in the dropout analyses due to the coding of the outcome variable (see above), leaving us with a final *n* = 1,447. The number of available cases for analyses varied substantially due to some missing data in the included variables (*n* varying between 1,065 and 1,229).

All analyses were conducted in IBM SPSS, version 25. Correlations were explored using Pearson’s r and Spearman’s rho for the continuous and dichotomous variables. We examined three simple associations through Chi square tests; one between health status (poor or good) and study track (vocational or general), one between health status (poor or good) and school status (dropout/completion), and one between study track and school status. All these variables are scored dichotomously; hence we report Phi coefficients as effect size measure.

Since dropout status is dichotomous a logistic regression analysis was used when examining predictors for drop out. Coefficients and effect sizes are reported by odds ratio (OR) and Nagelkerke’s *R*^2^ equivalent. Variables not contributing significantly (*p* > 0.10) were excluded backwardly. The predictors were organized in four blocks: (1) health-related variables, (2) demographics, (3) covariates, and (4) GPA. Within each block, except the final we removed variables with *p*-values above 0.10, to avoid excluding potentially relevant variables (Hosmer and Lemeshow, 2000). In the final block, only variables with *p*-values below 0.05 were retained. We also tested for interaction effects between mental health* track, general health*track, mental health*GPA, general health*GPA, mental health*sex and general health*sex. Finally, we tested how good the models were at classify cases correctly as dropouts or completers. The cut-off value was set to 0.327, corresponding to the observed share of dropouts in the sample.

## Results

Descriptive statistics and correlations between the variables are reported in Tables 1, 2. One third (33%) of the students had dropped out after 5 years, which was significantly (*p* < 0.001) related with several variables, the strongest associations being with lower GPA (*r* = −0.41), vocational track (*r* = −0.32) and literacy problems (*r* = 0.26).

**Table 1:** Descriptive statistics for all variables.

Variable (range)	N	Percent	M	SD	Median	Skewness	Kurtosis
1. Status (completer = 0; dropout = 1)	1,447	67.3/32.7					
2. SWLS (1–7)	1,569		3.19	1.37	3	0.57	−0.15
3a. HCSL-5 baseline (mean score ≤ 2 = 0; > 2 = 1)	1,608	82.6/16.7					
3b. HCSL-5 follow-up (mean score ≤ 2 = 0; > 2 = 1)	579	69.4/30.6					
4. Total sleep time workdays (hours)	1,509		6.81	1.24	7	−0.71	3.38
5. General health (0–2)	1,629		1.12	0.33	2	0.58	0.37
6. EDS (1–7)	1,628		2.55	1.29	2.2	1.10	0.64
7. SOC (1–7)	1,523		3.77	0.97	3.85	−0.13	−0.23
8. Sex (0 ♀. 1 ♂)	1,674	48/52					
9. Birthyear (94/95 = 1; older = 0)	1,674	83.7/16.3					
10. SES (1–4)	1,635		2.61	0.68	3	−0.10	−0.17
11. Social support (1–6)	1,636		4.45	0.58	4.5	−1.75	4.37
12. Bullying (1–6)	1,618		1.27	0.84	1	4.05	17.51
13. PAQ _masculine_ (1–5)	1,530		3.36	0.63	3.38	−0.23	0.27
14. PAQ _feminine_ (1–5)	1,530		3.79	0.67	3.88	−0.73	1.20
15. Promotion (1–5)	1,526		3.68	0.72	3.78	−0.79	1.54
16. Prevention (1–5)	1,526		3.05	0.70	3.11	−0.33	0.39
17. Literacy problems (0–1)	1,622		0.24	0.29	0.13	1.29	0.69
18. Plans for the future (not decided = 0; decided = 1)	1,674	26.3/73.7					
19. Track (vocational = 0; general = 1)	1,663	54.4/45.6					
20. GPA lower secondary (0–6)	1,355		4.09	0.80	4.13	−0.18	−0.49

SWLS, satisfaction with life scale; HSCL, Hopkins symptom checklist; EDS, eating disturbance scale; SOC, sense of coherence; SES, socioeconomic status; PAQ, personal attributes questionnaire.

**Table 2:** The association between the study variables as correlation coefficients.

Variable	1	2	3	4	5	6	7	8	9	10	11	12	13	14	15	16	17	18	19
1. Dropout status																			
2. SWLS	0.10^3^																		
3. HCSL	0.02	0.48^3^																	
4. Total sleep time workdays	−0.12^3^	−0.15^3^	−0.09^3^																
5. General health	0.14^3^	0.37^3^	0.33^3^	−0.17^3^															
6. EDS	−0.02	0.36^3^	0.42^3^	−0.12^3^	0.30^3^														
7. SOC (R)	0.08^2^	0.57^3^	0.58^3^	−0.20^3^	0.34^3^	0.46^3^													
8. Sex	0.17^3^	−0.11^3^	−0.20^3^	−0.05	−0.11^3^	−0.40^3^	−0.16^3^												
9. Birthyear	−0.09^3^	−0.15^3^	−0.12^3^	0.09^2^	−0.11^3^	−0.04	−0.09^3^	−0.03											
10. SES	−0.06^1^	−0.23^3^	−0.16^3^	0.10^3^	−0.19^3^	−0.06^1^	−0.17^3^	−0.02	0.09^3^										
11. Social support	−0.12^3^	−0.39^3^	−0.35^3^	0.09^3^	−0.24^3^	−0.17^3^	−0.36^3^	−0.11^3^	0.19^3^	0.19^3^									
12. Bullying	0.06^1^	0.14^3^	0.20^3^	−0.09^2^	0.08^3^	0.10^3^	0.17^3^	0.10^3^	−0.01	−0.05	−0.21^3^								
13. PAQ _masculine_	−0.12^3^	−0.43^3^	−0.32^3^	0.11^3^	−0.36^3^	−0.26^3^	−0.42^3^	0.09^3^	0.11^3^	0.25^3^	0.37^3^	−0.09^2^							
14. PAQ _feminine_	−0.12^3^	−0.15^3^	0.02	0.04	−0.09^2^	0.10^3^	−0.10^3^	−0.28^3^	0.10^3^	0.11^3^	0.27^3^	−0.08^2^	0.35^3^						
15. Promotion	−0.16^3^	−0.11^3^	0.05	0.14^3^	−0.15^3^	0.06^1^	−0.07^1^	−0.15^3^	−0.01	0.14^3^	0.15^3^	−0.10^3^	0.28^3^	0.33^3^					
16. Prevention	−0.04	0.25^3^	0.42^3^	−0.05	0.14^3^	0.31^3^	0.41^3^	−0.19^3^	−0.08^2^	−0.05^1^	−0.15^3^	0.09^2^	−0.22^3^	0.11^3^	0.42^3^				
17. Literacy problems	0.26^3^	0.11^3^	0.22^3^	−0.06^1^	0.20^3^	0.05	0.16^3^	0.15^3^	−0.21^3^	−0.15^3^	−0.24^3^	0.17^3^	−0.26^3^	−0.19^3^	−0.17^3^	0.10^3^			
18. Future plans	−0.04	−0.08^2^	−0.03	0.07^2^	−0.08^1^	−0.01	−0.07^2^	0.00	0.02	0.07^2^	0.09^3^	−0.02	0.19^3^	0.06^1^	0.12^3^	−0.03	−0.06^1^		
19. Track	−0.32^3^	−0.09^3^	−0.03	0.18^3^	−0.23^3^	−0.03	−0.13^3^	−0.14^3^	0.20^3^	0.14^3^	0.16^3^	−0.10^3^	0.23^3^	0.16^3^	0.26^3^	0.00	−0.29^3^	0.06^1^	
20. GPA lower secondary	−0.41^3^	−0.06^1^	−0.01	0.18^3^	−0.20^3^	0.08^2^	−0.08^2^	−0.29^3^	0.13^3^	0.14^3^	0.22^3^	−0.14^3^	0.26^3^	0.25^3^	0.31^3^	0.01	−0.44^3^	0.12^3^	0.59^3^

SWLS, satisfaction with life scale; HSCL-5, Hopkins symptom checklist – 5 items; EDS, eating disturbance scale; SOC, sence of coherence (reversed); SES, socioeconomic status; PAQ, personal attributes questionnaire; LP, literacy problems. Correlations are reported as Pearson’s r. *N* ranges from 1,192 (dropout vs. GPA) to 1,674 (gender vs. birthyear). ^3^ *p* < 0.001, ^2^ *p* < 0.01, and ^1^ *p* < 0.05.

The number of students with poor mental and general health was significantly higher in the vocational compared to the general track (Table 3). Furthermore, there was a significant difference between dropouts and completers within the vocational group regarding general health, but this difference was not present for mental health (Table 4). In the general track, students with good/poor self-reported mental and general health were comparably represented in the dropout and completers groups. A significantly higher number of students that enrolled in the general track completed upper secondary as compared to students enrolled in the vocational track (Table 5).

**Table 3:** Simple associations between health status and study track.

	Track		
Health status	Vocational^a^	General^a^	*p*
Mental health			
Good	694 (81%)	627 (85%)	
Poor	165 (19%)	113 (15%)	0.038
General health			
Good	724 (82%)	696 (94%)	
Poor	155 (18%)	45 (6%)	<0.001

a Percentage of total responders by track. *p* = chi-square probability value. Phi = −0.18, *p* < 0.001.

**Table 4:** Simple associations between health status and dropout among students in vocational track.

	School status		
Health status	Dropout	Completed	*p*
Mental health			
Good	263 (80%)	323 (83%)	
Poor	67 (20%)	66 (17%)	0.251
General health			
Good	270 (79%)	340 (86%)	
Poor	71 (21%)	56 (14%)	0.017

Respondents with unknown dropout status are excluded. *p* = chi-square probability value. Phi = −0.05, *p* < 0.001.

**Table 5:** Simple associations between school status and study track.

	Study track		
School Status	Vocational	General	*p*
Completed	404 (53.4%)	568 (83.4%)	
Dropout	352 (46.6%)	113 (16.6%)	<0.001

Respondents with unknown dropout status are excluded. *p* = chi-square probability value. Phi = −0.32, *p* < 0.001.

### Prediction of dropout

A logistic regression analysis was conducted to discern multivariable predictors of the five-year dropout rate (Table 6). The adjusted coefficients indicated that higher GPA from lower secondary school (OR = 0.31; *p* < 0.001) and being enrolled in the general track (OR = 0.66; *p* < 0.05) decreased the odds of dropping out as compared to lower GPA and vocational track students. The other variables that contributed significantly in the initial steps of the analysis, such as general health, EDS-5, SWLS and total sleep time, turned non-significant after the introduction of the GPA and the study track variables. Mental health (HSCL) did not contribute significantly neither in the unadjusted nor the adjusted analyses. The final model was statistically significant compared to a constant-only model, *χ*^2^ (7, *N* = 1,087) = 195.66, *p* < 0.001, accounting for 25% of the variance (Nagelkerke). The model classifies completers rather well (83% correct in the final model) but perform poorer on classifying dropouts (54%).

**Table 6:** Logistic regression analysis on predictors for dropout.

Blocks and variables	Block 1		Block 2		Block 3		Block 4		Predicted completed, dropped, total (%)^b^
	B (SE)	OR [95% CI]	B (SE)	OR [95% CI]	B (SE)	OR [95% CI]	B (SE)	OR [95% CI]	
Block 1: Health (*n* = 1,185)									80.7, 26.4, 67.8
Satisfaction with life	0.14 (0.05)	1.15** [1.04, 1.28]		ns					
Hopkins symptom checklist-5		ns							
Total sleep time workdays	−0.19 (0.05)	0.83*** [0.75, 0.92]	−0.13 (0.05)	0.88* [79, 0.97]		ns			
General health	0.69 (0.20)	1.99** [1.34, 2.97]	0.62 (0.21)	1.85** [1.22, 2.81]	0.40 (0.22)	1.50^a^ [0.98, 2.32]		ns	
Eating disturbance scale	−0.14 (0.06)	0.87* [0.78, 0.97]		ns		ns			
Sense of Coherence		ns							
Block 2: Demographics (*n* = 1,234)									87.0, 44.3, 74.3
Sex			0.66 (0.15)	1.94*** [1.44, 2.61]	0.55 (0.15)	1.73*** [1.29, 2.32]		ns	
Birth year			−1.77 (0.18)	0.17*** [0.12, 0.24]	−1.39 (0.20)	0.25*** [0.17, 0.37]		ns	
SES				ns					
Block 3: other variables (*n* = 1,160)									88.9, 40.1, 75.5
Support						ns			
Bullying						ns			
PAQ Masculine						ns			
PAQ Feminine						ns			
Promotion						ns			
Prevention					−0.23 (0.11)	0.80* [0.65, 0.98]			
Literacy problems					0.87 (0.28)	2.38** [1.38, 4.09]		ns	
Plans for further education						ns			
Track					−1.01 (0.16)	0.36*** [0.27, 50]	−0.42 (0.19)	0.66* [0.45, 0.96]	
Block 4 (*n* = 1,087)									83.2, 54.4, 76.5
GPA							−1.16 (0.15)	0.31*** [0.24, 0.42]	

SES, socio-economic status; PAQ, personal attributes questionnaire; GPA, grade point average. Nagelkerke R square = 0.24. Cut-off value is set to 0.327, corresponding to the observed share of dropouts in the sample. *** *p* < 0.001, ** *p* < 0.01, and * *p* < 0.05.

a The variable is non-significant, but with a *p*-value below 0.10 (see Data analyses section). ns, non-significant.

^b^ The percentage of correct classification of cases for each subsequent block/model. Cut-off value is set to 0.327, corresponding to the observed share of dropouts in the sample.

In order to examine if the nonsignificant mental health – dropout relationship could be masked by other factors, we added a range of interaction terms to the logistic model: mental health* track, general health*track, mental health*GPA, general health*GPA, mental health*sex and general health*sex. Even though the initial descriptive statistics showed some health differences among vocational and general track, none of these variables moderated the primary relationship significantly when included as interaction terms in the full analyses. Hence, we did not find support for the existence of any sub-group relationships between health and dropout status.

A follow up data collection was conducted 30 months after baseline, but the attrition of participants was very high (*N* = 661, 39% participated). Attrition analyses revealed that the participants and the non-participants in the follow-up study where significantly different from each other on group level analyses on demographic baseline variables. The participants and non-participants were different with regards to gender distribution (χ^2^ (1, *N* = 661) = 59.20, *p* < 0.001, *Phi* = −0.19) and track distribution (χ^2^ (2, *N* = 661) = 12.71, *p* < 0.01, *Phi* = 0.09), where more females and students in general track participated in the follow up study. The attrition group was also different from the follow-up group on high school GPA (*t* = −7.47, *df* (1353), *p* < 0.001), where non-participants had lower GPA (M: 39.57, SD: 7.82) than participants (M: 42.78, SD: 7.76).

This, coupled with missing data on the predictor and outcome variables, a regression analysis including both time points was not deemed valid.

We did nevertheless examine if health status worsened during the study years by examining stability and change in the health variables among the students answering both data collections. The proportion of students increasing their HSCL-score above the cut-off (>2) was 22%, whereas those crossing below the cut-off was 34%. Because more students changed their health status in a favourable rather than unfavourable direction, the lack of significant predictive findings for health status rather strengthen than weaken the validity of this null-finding.

## Discussion

The aim of this study was to investigate a collection of academic, psychosocial, and health-related predictors of dropout from upper secondary school. We expected that health-related variables (satisfaction with life, sleep habits, general health, internalized mental health problems, eating disturbances and sense of coherence) would predict dropout 5 years later, as well as social support, bullying, personality, motivational goals, ambitions, and literacy problems. In addition, we expected that being male, a student older than the typical age, enrolled in vocational track, and having poorer grades would contribute to predicting dropout. Finally, we aimed to investigate these variables through a follow-up study, but due to high attrition, we did not include data from the second data collection in the dropout analyses.

In the first analyses of simple associations, we found that both mental and general health were poorer among students in vocational track than among students in general track. We also found that general health was poorer among students that had left school, and that dropout rates were higher among students enrolled in the vocational track. In addition, dropout was negatively associated with GPA.

However, when entering other variables in the analyses, all the health variables turned non-significant. Also, demographics and other variables were non-significant in the final model, leaving track and GPA as the only significant predictors for dropout. However, these effect sizes were small.

Contrary to other studies (Hjorth et al., 2016; Askeland et al., 2022; Lindhardt et al., 2022), we did not find a clear association between mental health and dropout, but the study *does* confirm previous findings indicating that lower grades (e.g., Allensworth and Easton, 2005; Bowers, 2010; Casillas et al., 2012) predict school dropout. A meta-analysis from 2014 (Esch et al., 2014) indicated an association between measures of emotional problems and dropout (*r* = 0.15–0.18); however, these associations were of a small magnitude even in unadjusted analyses. Another study by Sagatun et al. (2014), who adjusted for grades, did not observe a significant relationship. In a review of school related variables and mental health, Gustafsson et al. (2010) reported associations between internalizing problems and poor academic achievement, which in turn is known to be associated with dropout (Bowers, 2010; Casillas et al., 2012). Taken together, we conclude that internalized mental health at the start of upper secondary school is not the most important factor for dropping out of school given the existing null-findings, or in best case, findings of minor effect sizes.

The transition between lower and upper secondary, is a vulnerable phase (Bowers, 2010). In our study, we collected the data within the first 2 months of upper secondary school. Fluctuation in the individual symptom burden related to mental health (Fleming et al., 2014; WHO, 2017), which may be particularly present in the beginning of the high school years, may underpin the lack of a predictive contribution by self-rated mental health in our analyses. The fact that the students were trying to adapt to a new environment could inflict higher levels of anxiety or worry during the first months, not representing their stable condition. Moreover, if students potentially improved their mental health status during the study period, these favourable changes may have aroused feelings of hope and positive expectations related to a new start. Another possibility is that an ongoing drop-out process may be associated with negative feelings, less efficient or unhelpful emotional regulatory strategies that were not present when beginning upper secondary school (Ottosen et al., 2017).

Bania et al. (2016) found that worse mental health predicted dropout exclusively among females, and boys dominated the vocational track where the dropout rates were highest. Previous research has indicated that boys report a lower symptom burden than girls (Sigmon et al., 2005; Bramness et al., 2010; Krokstad et al., 2022), but our data provided no indication of sex as a moderator between symptom burden and dropout.

When it comes to self-reported general health, De Ridder et al. (2012) has shown an association with school-dropout. However, they did not control for variables directly related to school, like track or grades. In our study, general health was a significant predictor initially, but as soon as other predictors were included in the analyses, health was no longer an independent predictor. Even though the effects are small, grades and track seem better at predicting dropout than health.

In line with the national statistics for dropout (Statistics Norway, 2023) males had a higher risk of dropping out than females initially in the regression analyse; however, this difference disappears when we adjust for grades. This could relate to the fact that males often achieve poorer academically than females (Markussen et al., 2011; Sæle et al., 2016).

In our study we found that mental health screening in the beginning of upper secondary school is not enough to predict later dropout. However, Dupéré et al. (2018) found in a retrospective study conducted shortly after dropout of secondary education, that nearly 25% of them had clinical depressive symptoms within the last 3 months before leaving. Many adolescent struggles in school and in life as well. This may have implications for the healthcare services provided for youth, that is the threshold for receiving psychosocial healthcare services at the schools should be low. Health care workers may help to identify students at risk of poor mental health (Gall et al., 2000; Walker et al., 2010) which, as shown in other studies reduces the risk of dropout (Kerns et al., 2011; Westbrook et al., 2020).

In summary, our data did not confirm our hypothesis that general health, mental health, sense of coherence, life satisfaction, sleep habits or eating disturbances or even sex predicts dropout. However, being enrolled at a vocational track and having achieved lower GPA from lower secondary education do, to some degree, predict dropout.

### Strengths and limitations of the study

Strengths of study: The sample size was fairly large (*N* = 1,676) and also quite representative for the entire cohort in this North-Norwegian county due to a high participation rate (69%). This response rate is clearly above what is normally expected in general epidemiological studies as of today (Hysing et al., 2013; Hjorth et al., 2016). The comprehensive baseline characterization and the minor prospective part of the current study are also strengths.

Limitations of the study: While the aim of this particular study was to focus on health variables, the complete survey “ung vilje,” consisted of a broad set of questions and instruments. Because the study was exploratory, and because the original data collection happened in 2010, the hypotheses are not pre-registered. Use of comprehensive questionnaire may incur limitations, by tiring students before reaching the final questions, leading to less valid responses of items (Sahlqvist et al., 2011). In “ung vilje” the health questions appeared early in the questionnaire leaflet (first one-third), which may have reduced this risk.

Self-reported data can lead to misreporting; the questions may be misunderstood or to complex and by using validated instruments previously used in similar populations this risk is minimized (Boynton and Greenhalgh, 2004).

The data were collected only a few weeks after the students entered first year in upper secondary school, and their responses to some of the questions may primarily have reflected previous school experience or the change of school *per se.*

Despite the large original sample size (1674), a relatively large number of students had to be excluded because of missing values on GPA or missing status of completion. Together with missing responses, this left us with 1,187 at the final stage in the logistic regression analysis.

### Recommendation for future research

The relationships between dropout and sex, track, mental and general health, and grades still need further investigation. The symptom burden among students should be measured at several time points throughout the whole study period, in order to better identify and elucidate the possible relationships between mental and general health and dropout. Whether differences in self-rated mental health between boys and girls are due to real diversity or to under-reported symptoms are also questions to be further investigated.

## Conclusion

Low grades are suited to function as a warning flag for dropout, as others have suggested earlier. Further studies should therefor make sure to control for grades, when investigating other variables. Our findings indicate that general health is associated with dropout among students in the vocational track, but not when controlling for previous grades. This result needs replication and further investigation.

Internalized mental problems in the beginning of upper secondary school do not predict dropout. However, the students report of mental health is changing during adolescence, and reports from the beginning of upper secondary may be influenced by expectations and optimism related to a new start. It may therefore still be important to monitor the development of the mental health condition of the individual student during the whole study period.

## Data availability statement

The original contributions presented in the study are publicly available. This data can be found here: https://dataverse.no/dataset.xhtml?persistentId=doi:10.18710/Q3GFGG.

## Ethics statement

The studies involving humans were approved by Regional Comittee for Medical Research Ethics Northern Norway. The studies were conducted in accordance with the local legislation and institutional requirements. Written informed consent for participation in this study was provided by the participants’ legal guardians/next of kin.

## Author contributions

CG: Conceptualization, Data curation, Formal analysis, Funding acquisition, Investigation, Methodology, Project administration, Writing – original draft. TS: Conceptualization, Funding acquisition, Methodology, Project administration, Supervision, Writing – review & editing. OF: Conceptualization, Methodology, Supervision, Writing – review & editing. KO: Conceptualization, Data curation, Funding acquisition, Writing – review & editing. RS: Data curation, Formal analysis, Methodology, Supervision, Writing – review & editing.

## References

[ref1] Allen K. L. Byrne S. M. Oddy W. H. Crosby R. D. (2013). DSM–IV–TR and DSM-5 eating disorders in adolescents: prevalence, stability, and psychosocial correlates in a population-based sample of male and female adolescents. J. Abnorm. Psychol. 122, 720–732. doi: 10.1037/a0034004 24016012

[ref2] Allensworth E. Easton J. Q. (2005) The on-track indicator as a predictor of high school graduation. Consortium on Chicago School Research at the University of Chicago. Available at: https://search.issuelab.org/resources/1/1.pdf.

[ref3] Antonovsky A. (1993). The structure and properties of the sense of coherence scale. Soc. Sci. Med. 36, 725–733. doi: 10.1016/0277-9536(93)90033-Z 8480217

[ref4] Askeland K. G. Bøe T. Sivertsen B. Linton S. J. Heradstveit O. Nilsen S. A.. (2022). Association of depressive symptoms in late adolescence and school dropout. Sch. Ment. Heal. 14, 1044–1056. doi: 10.1007/s12310-022-09522-5

[ref5] Bania E. V. Lydersen S. Kvernmo S. (2016). Non-completion of upper secondary school among female and male young adults in an Arctic sociocultural context; the NAAHS study. BMC Public Health 16:960. doi: 10.1186/s12889-016-3644-2, PMID: 27618990 PMC5020482

[ref6] Bøe T. Øverland S. Lundervold A. J. Hysing M. (2012). Socioeconomic status and children’s mental health: results from the Bergen child study. Soc. Psychiatry Psychiatr. Epidemiol. 47, 1557–1566. doi: 10.1007/s00127-011-0462-9 22183690

[ref7] Bowers A. J. (2010). Grades and graduation: a longitudinal risk perspective to identify student dropouts. J. Educ. Res. 103, 191–207. doi: 10.1080/00220670903382970

[ref8] Boynton P. M. Greenhalgh T. (2004). Hands-on guide to questionnaire research: selecting, designing, and developing your questionnaire, BMJ. Br. Med. J. 328, 1312–1315. doi: 10.1136/bmj.328.7451.1312, PMID: 15166072 PMC420179

[ref9] Bramness J. G. Walby F. A. Hjellvik V. Selmer R. Tverdal A. (2010). Self-reported mental health and its gender differences as a predictor of suicide in the middle-aged. Am. J. Epidemiol. 172, 160–166. doi: 10.1093/aje/kwq091, PMID: 20519262

[ref10] Breidablik H. J. Meland E. (2001). Ung på hybel – sosial kontroll og helserelatert atferd. Tidsskr. Nors. Legeforen. 121, 287–291. Available at: https://tidsskriftet.no/2001/01/klinikk-og-forskning/ung-pa-hybel-sosial-kontroll-og-helserelatert-atferd 11242867

[ref11] Breidablik H.-J. Meland E. Lydersen S. (2009). Self-rated health during adolescence: stability and predictors of change (young-HUNT study, Norway). Eur. J. Public Health 19, 73–78. doi: 10.1093/eurpub/ckn111, PMID: 19022851 PMC2639013

[ref12] Casillas A. Robbins S. Allen J. Kuo Y. L. Hanson M. A. Schmeiser C. (2012). Predicting early academic failure in high school from prior academic achievement, psychosocial characteristics, and behavior. J. Educ. Psychol. 104, 407–420. doi: 10.1037/a0027180

[ref13] Curnock E. Leyland A. H. Popham F. (2016). The impact on health of employment and welfare transitions for those receiving out-of-work disability benefits in the UK. Soc. Sci. Med. 162, 1–10. doi: 10.1016/j.socscimed.2016.05.042, PMID: 27318626 PMC4962812

[ref14] Dæhlen M. (2017). Completion in vocational and academic upper secondary school: the importance of school motivation, self-efficacy, and individual characteristics. Eur. J. Educ. 52, 336–347. doi: 10.1111/ejed.12223

[ref15] De Ridder K. A. A. Pape K. Cuypers K. Johnsen R. Holmen T. L. Westin S.. (2013). High school dropout and long-term sickness and disability in young adulthood: a prospective propensity score stratified cohort study (the young-HUNT study). BMC Public Health 13:941. doi: 10.1186/1471-2458-13-941, PMID: 24103558 PMC4124891

[ref16] De Ridder K. A. A. Pape K. Johnsen R. Westin S. Holmen T. L. Bjørngaard J. H. (2012). School dropout: a major public health challenge: a 10-year prospective study on medical and non-medical social insurance benefits in young adulthood, the young-HUNT 1 study (Norway). J. Epidemiol. Community Health 66, 995–1000. doi: 10.1136/jech-2011-200047, PMID: 22315238

[ref17] Derdikman-Eiron R. Indredavik M. S. Bratberg G. H. Taraldsen G. Bakken I. J. Colton M. (2011). Gender differences in subjective well-being, self-esteem and psychosocial functioning in adolescents with symptoms of anxiety and depression: findings from the Nord-Trondelag health study. Scand. J. Psychol. 52, 261–267. doi: 10.1111/j.1467-9450.2010.00859.x, PMID: 21265857

[ref18] Diener E. Emmons R. A. Larsen R. J. Griffin S. (1985). The satisfaction with life scale. J. Pers. Assess. 49, 71–75. doi: 10.1207/s15327752jpa4901_13 16367493

[ref19] Dupéré V. Dion E. Nault-Brière F. Archambault I. Leventhal T. Lesage A. (2018). Revisiting the link between depression symptoms and high school dropout: timing of exposure matters. J. Adolesc. Health 62, 205–211. doi: 10.1016/j.jadohealth.2017.09.024, PMID: 29195763

[ref20] Dweck C. S. (1986). Motivational processes affecting learning. Am. Psychol. 41, 1040–1048. doi: 10.1037/0003-066X.41.10.1040

[ref21] Esch P. Bocquet V. Pull C. Couffignal S. Lehnert T. Graas M.. (2014). The downward spiral of mental disorders and educational attainment: a systematic review on early school leaving. BMC Psychiatry 14:237. doi: 10.1186/s12888-014-0237-4, PMID: 25159271 PMC4244046

[ref22] Fleming T. M. Clark T. Denny S. Bullen P. Crengle S. Peiris-John R.. (2014). Stability and change in the mental health of New Zealand secondary school students 2007–2012: results from the national adolescent health surveys. Aust. N. Z. J. Psychiatry 48, 472–480. doi: 10.1177/0004867413514489, PMID: 24317154

[ref23] Gall G. Pagano M. E. Desmond M. S. Perrin J. M. Murphy J. M. (2000). Utility of psychosocial screening at a school-based health center. J. Sch. Health 70, 292–298. doi: 10.1111/j.1746-1561.2000.tb07254.x, PMID: 10981284 PMC3306214

[ref24] Goodman E. Adler N. E. Kawachi I. Frazier A. L. Huang B. Colditz G. A. (2001). Adolescents' perceptions of social status: development and evaluation of a new indicator. Pediatrics 108:e31. doi: 10.1542/peds.108.2.e31, PMID: 11483841

[ref25] Gubbels J. van der Put C. E. Assink M. (2019). Risk factors for school absenteeism and dropout: a meta-analytic review. J. Youth Adolesc. 48, 1637–1667. doi: 10.1007/s10964-019-01072-5, PMID: 31312979 PMC6732159

[ref26] Gustafsson J.-E. Allodi Westling M. Alin Åkerman B. Eriksson C. Eriksson L. Fischbein S.. (2010) School, learning and mental health: a systematic review. 978-91-7190-138-5 (ISBN). Stockholm: Kungl. Vetenskapsakademien. 187 + 36 s. Available at: https://su.diva-portal.org/smash/get/diva2:317965/FULLTEXT01.pdf.

[ref27] Hale D. R. Viner R. M. (2018). How adolescent health influences education and employment: investigating longitudinal associations and mechanisms. J. Epidemiol. Community Health 72, 465–470. doi: 10.1136/jech-2017-209605, PMID: 29615474 PMC5969389

[ref28] Halvorsrud K. (2017). Student dropout in upper secondary education in Norway: a challenge to the principles of the welfare state? London Review of Education, 15. London: UCL Institute of Education, 302–316 s.

[ref29] Helmreich R. Spence J. Wilhelm J. (1981). A psychometric analysis of the personal attributes questionnaire. Sex Roles 7, 1097–1108. doi: 10.1007/BF00287587

[ref30] Heradstveit O. Holmelid E. Klundby H. Søreide B. Sivertsen B. Sand L. (2019). Associations between symptoms of eating disturbance and frequency of physical activity in a non-clinical, population-based sample of adolescents. J. Eat. Disord. 7:9. doi: 10.1186/s40337-019-0239-1, PMID: 31019696 PMC6471893

[ref31] Hetlevik Ø. Smith-Sivertsen T. Haukenes I. Ruths S. Baste V. (2023). Young adults with depression: a registry-based longitudinal study of work-life marginalisation. The Norwegian GP-DEP study. Scand. J. Public Health. doi: 10.1177/14034948231165089 PMC11292960 37066887

[ref32] Higgins E. T. Fowler R. D. (1997). Beyond pleasure and pain. Am. Psychol. 52, 1280–1300. doi: 10.1037/0003-066X.52.12.1280 9414606

[ref33] Hjorth C. F. Bilgrav L. Frandsen L. S. Overgaard C. Torp-Pedersen C. Nielsen B.. (2016). Mental health and school dropout across educational levels and genders: a 4.8-year follow-up study. BMC Public Health 16:976. doi: 10.1186/s12889-016-3622-8, PMID: 27627885 PMC5024430

[ref34] Hosmer D. W. Lemeshow S. (2000) Applied logistic regression. 2nd ed. New York, Toronto: Wiley.

[ref35] HUNT (2013). Available at: http://www.ntnu.no/hunt/skjema.

[ref36] Hysing M. Pallesen S. Stormark K. M. Lundervold A. J. Sivertsen B. (2013). Sleep patterns and insomnia among adolescents: a population-based study. J. Sleep Res. 22, 549–556. doi: 10.1111/jsr.12055 23611716

[ref37] Idler E. L. Benyamini Y. (1997). Self-rated health and mortality: a review of twenty-seven community studies. J. Health Soc. Behav. 38, 21–37. doi: 10.2307/2955359 9097506

[ref38] Irvin M. J. Farmer T. W. Weiss M. P. Meece J. L. Byun S. Y. McConnell B. M.. (2011). Perceptions of school and aspirations of rural students with learning disabilities and their nondisabled peers. Learn. Disabil. Res. Pract. 26, 2–14. doi: 10.1111/j.1540-5826.2010.00320.x

[ref39] Kerns S. E. U. Pullmann M. D. Walker S. C. Lyon A. R. Cosgrove T. J. Bruns E. J. (2011). Adolescent use of school-based health centers and high school dropout. Arch. Pediatr. Adolesc. Med. 165, 617–623. doi: 10.1001/archpediatrics.2011.10, PMID: 21383256

[ref40] Korhonen J. Linnanmäki K. Aunio P. (2014). Learning difficulties, academic well-being and educational dropout: a person-centred approach. Learn. Individ. Differ. 31, 1–10. doi: 10.1016/j.lindif.2013.12.011

[ref41] Kortering L. J. Braziel P. M. McClannon T. W. (2010). Career ambitions: a comparison of youth with and without SLD. Remedial Spec. Educ. 31, 230–240. doi: 10.1177/0741932508324404

[ref42] Kouvonen A. M. Vaananen A. Vahtera J. Heponiemi T. Koskinen A. Cox S. J.. (2010). Sense of coherence and psychiatric morbidity: a 19-year register-based prospective study. J. Epidemiol. Community Health 64, 255–261. doi: 10.1136/jech.2008.083352, PMID: 19706620

[ref43] Krokstad S. Weiss D. A. Krokstad M. A. Rangul V. Kvaløy K. Ingul J. M.. (2022). Divergent decennial trends in mental health according to age reveal poorer mental health for young people: repeated cross-sectional population-based surveys from the HUNT study, Norway. BMJ Open 12:e057654. doi: 10.1136/bmjopen-2021-057654, PMID: 35584877 PMC9119156

[ref44] Lindhardt L. Lindholdt L. Lund T. Mortensen O. S. (2022). Self-reported mental health in adolescents attending school and its association with later school dropout: a prospective 2.5-year follow-up study. Scand. J. Public Health 50, 1164–1171. doi: 10.1177/14034948221089112, PMID: 35441561

[ref45] Lockwood P. Jordan C. H. Kunda Z. (2002). Motivation by positive or negative role models: regulatory focus determines who will best inspire us. J. Pers. Soc. Psychol. 83, 854–864. doi: 10.1037/0022-3514.83.4.854 12374440

[ref46] Markussen E. (2017). Education pays off! On transition to work for 25 year olds in Norway with upper secondary education or lower as their highest educational level. Educ. Res. Policy Prac. 16, 27–42. doi: 10.1007/s10671-016-9201-z

[ref47] Markussen E. Frøseth M. W. Sandberg N. Lødding B. Borgen J. S. (2011) Early leaving, non-completion and completion in upper secondary education in Norway, In Lamb S. Markussen E. Teese R. Sandberg N. Polesel J. (eds.) School dropout and completion: international comparative studies in theory and policy. Dordrecht: Springer Netherlands, 253–271.

[ref48] McLaughlin M. J. Speirs K. E. Shenassa E. D. (2014). Reading disability and adult attained education and income: evidence from a 30-year longitudinal study of a population-based sample. J. Learn. Disabil. 47, 374–386. doi: 10.1177/0022219412458323 22983608

[ref49] Mittelmark M. B. Sagy S. Eriksson M. Bauer G. F. Pelikan J. M. Lindström B.. (eds.) (2017). The handbook of Salutogenesis. Switzerland: Springer International Publishing. 28590610

[ref50] Moksnes U. K. Espnes G. A. Haugan G. (2014). Stress, sense of coherence and emotional symptoms in adolescents. Psychol. Health 29, 32–49. doi: 10.1080/08870446.2013.822868, PMID: 23906224

[ref51] Moksnes U. K. Løhre A. Espnes G. A. (2013). The association between sense of coherence and life satisfaction in adolescents. Qual. Life Res. 22, 1331–1338. doi: 10.1007/s11136-012-0249-9, PMID: 22886139

[ref52] Myklestad I. Røysamb E. Tambs K. (2012). Risk and protective factors for psychological distress among adolescents: a family study in the Nord-Trøndelag health study. Soc. Psychiatry Psychiatr. Epidemiol. 47, 771–782. doi: 10.1007/s00127-011-0380-x, PMID: 21499806

[ref53] NAV (2023a) Diagnoser uføretrygd. Available at: https://www.nav.no/no/nav-og-samfunn/statistikk/aap-nedsatt-arbeidsevne-og-uforetrygd-statistikk/uforetrygd/diagnoser-uforetrygd_kap.

[ref54] NAV (2023b) Uføretrygd – kvartalsstatistikk. Available at: https://www.nav.no/no/nav-og-samfunn/statistikk/aap-nedsatt-arbeidsevne-og-uforetrygd-statistikk/uforetrygd/statistikknotis-uforetrygd-utviklingen-per-mars-2023.

[ref55] NIPH (2018) Public health report: health status in Norway 2018: Norwegian Institute of Public Health Oslo. Available at: https://www.fhi.no/en/publ/2018/health-status-in-norway-2018/.

[ref56] Norwegian Directorate for Education and Training (2023) The Norwegian education Mirror 2022. The Norwegian Directorate for Education and Training. Available at: https://www.udir.no/in-english/the-education-mirror-2022/upper-secondary-school-education/.

[ref57] OECD (2022). Education at a glance 2022. OECD indicators. Paris: OECD Publishing.

[ref58] Ottosen K. O. Goll C. B. Sørlie T. (2017). “From a sense of failure to a proactive life orientation”: first year high school dropout experiences and future life expectations in Norwegian youth. Int. Soc. Work. 62, 684–698. doi: 10.1177/0020872817746225

[ref59] Patel V. Flisher A. J. Hetrick S. McGorry P. (2007). Mental health of young people: a global public-health challenge. Lancet 369, 1302–1313. doi: 10.1016/S0140-6736(07)60368-7 17434406

[ref60] Pavot W. Diener E. Butcher J. N. (1993). Review of the satisfaction with life scale. Psychol. Assess. 5, 164–172. doi: 10.1037/1040-3590.5.2.164

[ref61] Reid G. Holt M. K. Bowman C. E. Espelage D. L. Green J. G. (2016). Perceived social support and mental health among first-year college students with histories of bullying victimization. J. Child Fam. Stud. 25, 3331–3341. doi: 10.1007/s10826-016-0477-7

[ref62] Roenneberg T. Kuehnle T. Pramstaller P. P. Ricken J. Havel M. Guth A.. (2004). A marker for the end of adolescence. Curr. Biol. 14, R1038–R1039. doi: 10.1016/j.cub.2004.11.039 15620633

[ref63] Roenneberg T. Wirz-Justice A. Merrow M. (2003). Life between clocks: daily temporal patterns of human chronotypes. J. Biol. Rhythm. 18, 80–90. doi: 10.1177/0748730402239679, PMID: 12568247

[ref64] Rosenvinge J. H. Perry J. A. Bjørgum L. Bergersen T. D. Silvera D. H. Holte A. (2001). A new instrument measuring disturbed eating patterns in community populations: development and initial validation of a five-item scale EDS-5. Eur. Eat. Disord. Rev. 9, 123–132. doi: 10.1002/erv.371

[ref65] Rumberger R. W. Lim S. A. (2008) Why students drop out of school: a review of 25 years of research. California: University of California, Santa Barbara. Available at: https://iris.who.int/bitstream/handle/10665/254610/W?sequence=1.

[ref66] Sæle R. G. Sørlie T. Nergård-Nilssen T. Ottosen K. O. Goll C. B. Friborg O. (2016). Demographic and psychological predictors of grade point average (GPA) in North-Norway: a particular analysis of cognitive/school-related and literacy problems. Educ. Psychol. 36, 1886–1907. doi: 10.1080/01443410.2014.998630

[ref67] Sagatun Å. Heyerdahl S. Wentzel-Larsen T. Lien L. (2014). Mental health problems in the 10thgrade and non-completion of upper secondary school: the mediating role of grades in a population-based longitudinal study. BMC Public Health 14:16. doi: 10.1186/1471-2458-14-16, PMID: 24406098 PMC3905670

[ref68] Sahlqvist S. Song Y. Bull F. Adams E. Preston J. Ogilvie D.. (2011). Effect of questionnaire length, personalisation and reminder type on response rate to a complex postal survey: randomised controlled trial. BMC Med. Res. Methodol. 11:62. doi: 10.1186/1471-2288-11-62, PMID: 21548947 PMC3110121

[ref69] Sigmon S. T. Pells J. J. Boulard N. E. Whitcomb-Smith S. Edenfield T. M. Hermann B. A.. (2005). Gender differences in self-reports of depression: the response bias hypothesis revisited. Sex Roles 53, 401–411. doi: 10.1007/s11199-005-6762-3

[ref70] Spence J. T. Helmreich R. L. (1978) Masculinity & femininity: their psychological dimensions, correlates, & antecedents. Austin: University of Texas Press.

[ref71] Statistics Norway (2023) Completion rates of pupils in upper secondary education. 12.06.2023 utg.: SSB. Available at: https://www.ssb.no/en/utdanning/videregaende-utdanning/statistikk/gjennomforing-i-videregaende-opplaering.

[ref72] Strand B. H. Dalgard O. S. Tambs K. Rognerud M. (2003). Measuring the mental health status of the Norwegian population: a comparison of the instruments SCL-25, SCL-10, SCL-5 and MHI-5 (SF-36). Nord. J. Psychiatry 57, 113–118. doi: 10.1080/08039480310000932, PMID: 12745773

[ref73] Super S. Verschuren W. M. M. Zantinge E. M. Wagemakers M. A. E. Picavet H. S. J. (2014). A weak sense of coherence is associated with a higher mortality risk. J. Epidemiol. Community Health 68, 411–417. doi: 10.1136/jech-2013-203085, PMID: 24385549

[ref74] Tambs K. Moum T. (1993). How well can a few questionnaire items indicate anxiety and depression? Acta Psychiatr. Scand. 87, 364–367. doi: 10.1111/j.1600-0447.1993.tb03388.x, PMID: 8517178

[ref75] Thern E. de Munter J. Hemmingsson T. Rasmussen F. (2017). Long-term effects of youth unemployment on mental health: does an economic crisis make a difference? J. Epidemiol. Community Health 71, 344–349. doi: 10.1136/jech-2016-208012, PMID: 28087812 PMC5484029

[ref76] Tromsøundersøkelsen (2013) The Tromsø Study / Tromsøundersøkelsen. Available at: http://uit.no/ansatte/organisasjon/artikkel?p_document_id=70715&p_dimension_id=88111&p_menu=42515.

[ref77] Vilbli (2024) Vilbli.no. Available at: https://www.vilbli.no/nb/nb/no/hva-er-videregaende-opplaering/a/035076.

[ref78] Wagner M. Newman L. Cameto R. Garza N. Levine P. (2005) After high school: a first look at the postschool experiences of youth with disabilities. A report from the National Longitudinal Transition Study-2 (NLTS2). Washington: Office of Special Education Programs U.S. Department of Education. Available at: https://files.eric.ed.gov/fulltext/ED494935.pdf.

[ref79] Walker S. C. Kerns S. E. U. Lyon A. R. Bruns E. J. Cosgrove T. J. (2010). Impact of school-based health center use on academic outcomes. J. Adolesc. Health 46, 251–257. doi: 10.1016/j.jadohealth.2009.07.002 20159502

[ref80] Westbrook M. Martinez L. Mechergui S. Yeatman S. (2020). The influence of school-based health center access on high school graduation: evidence from Colorado. J. Adolesc. Health 67, 447–449. doi: 10.1016/j.jadohealth.2020.04.012, PMID: 32532565 PMC7483255

[ref81] WHO. (2013a) The economics of social determinants of health and health inequalities. Available at: http://apps.who.int/iris/bitstream/handle/10665/84213/9789241548625_eng.pdf?sequence=1.

[ref82] WHO. (2013b) Mental health action plan 2013 – 2020. Available at: https://apps.who.int/iris/bitstream/handle/10665/89966/9789241506021_eng.pdf;jsessionid=67E794742482EFC1578FB76B661D65F8?sequence=1.

[ref83] WHO. (2017) Depression and other common mental disorders: Global health estimates. Geneva: World Health Organization. Available at: https://iris.who.int/bitstream/handle/10665/254610/W?sequence=1.

[ref84] WHO. (2021) Mental health of adolescents. Available at: https://www.who.int/news-room/fact-sheets/detail/adolescent-mental-health.

[ref85] Zhang J. Paksarian D. Lamers F. Hickie I. B. He J. Merikangas K. R. (2017). Sleep patterns and mental health correlates in US adolescents. J. Pediatr. 182, 137–143. doi: 10.1016/j.jpeds.2016.11.007 27939122

